# Experiences of simulated patients in clinical skills laboratory: A qualitative study

**DOI:** 10.4102/curationis.v48i1.2736

**Published:** 2025-09-26

**Authors:** Mbalenhle P. Shange-Goba, Juliana J. Willemse

**Affiliations:** 1 School of Nursing, Faculty of Community and Health Sciences, University of the Western Cape, Cape Town, South Africa

**Keywords:** simulated patients, nursing education, clinical skills laboratory, qualitative research, simulation experiences

## Abstract

**Background:**

Nursing simulations with simulated patients (SPs) have proven effective in creating realistic opportunities to enhance the students’ clinical competence within a safe learning environment. This setting helps to reduce anxiety and increases self-confidence among health sciences students. This study aimed to explore experiences of SPs in clinical skills laboratory of a Department of Nursing at a university in Western Cape.

**Objectives:**

Objectives were set to explore the contextual elements of SPs’ experiences during clinical skills sessions, identify educational design aspects from those experiences and assess their views on student outcomes.

**Method:**

A qualitative descriptive, exploratory and contextual design was used. Data were collected through semi-structured interviews with eight SPs. The data were coded to develop emerging themes and sub-themes, following Braun and Clark’s systematic analysis process. An independent coder reviewed findings, and themes were confirmed during a consensus meeting.

**Results:**

This study found that SPs engaged in multiple role-play consultations, improving their understanding of healthcare consultation structures and rules. They recognised their roles in achieving the outcomes needed for students.

**Conclusion:**

This study confirmed existing literature on SPs in a clinical skills laboratory within a Department of Nursing. Participant interviews provided verbatim quotations that enriched findings.

**Contribution:**

The study aimed to recommend ways for a Department of Nursing to support SPs in improving their laboratory skills and enhancing student learning. Expanding this research to other nursing education institutions could provide a broader understanding of SPs’ experiences.

## Introduction

Simulation is an educational strategy that offers students realistic clinical situations to practice and learn clinical skills in a safe learning environment (Goba 2022; MacLean et al. 2023; Wong, Print & Gerzina 2019). By integrating simulation into nursing education, students can develop their clinical skills in a non-threatening environment where mistakes can be made without causing harm to patients (Alshehri et al. 2023; Koukourikos et al. 2021). Current research shows that this safe environment significantly reduces anxiety and increases self-confidence among health sciences students, with some studies reporting an improvement of up to 45% following exposure to simulations (Anderson et al. 2023; Williams 2024).

The evolution of simulation in health sciences education has been characterised by significant technological advances and pedagogical innovations over the past decade (Thompson & Martinez 2023). Modern simulation laboratories employ various modalities, including high-fidelity mannequins, virtual reality platforms and trained simulated patients (SPs), creating a comprehensive learning environment that addresses multiple learning objectives simultaneously (Chen & Rodriguez 2024). These advanced simulation technologies have transformed the way clinical skills are taught, enabling more precise feedback and assessment of student performance (Wilson & Kumar 2024).

Research indicates that simulation-based education greatly enhances critical thinking, clinical reasoning and decision-making skills among healthcare students (Martinez & Thompson 2024; Msosa et al. 2024). Studies demonstrate that students who participate in regular simulation sessions achieve clinical performance scores that are, on average, 37% higher than those who receive only traditional clinical education (Davidson, Howell & Davenport 2023). In addition, simulation-based training has been shown to reduce medical errors in clinical practice by up to 28% (Park & Anderson 2024).

The incorporation of SPs into healthcare education has emerged as an especially effective element of comprehensive simulation programmes (Hassan & Lee 2023). The SPs provide authentic human interactions that help students develop essential communication and interpersonal skills while learning clinical procedures (Wilson, Thompson & Rodriguez 2024). Recent studies indicate that training involving SPs led to significantly higher patient satisfaction scores when students transition to real clinical settings, with improvements ranging from 32% to 45% compared to traditional training methods (Kumar & Rodriguez 2023).

This effective learning approach allows nursing students to integrate theory with practice, facilitating the transfer of knowledge from simulated settings into real-life situations (Thompson & Martinez 2023; Zhang et al. 2024). Contemporary research highlights that simulation-based education enhances clinical competency, improves awareness of patient safety and strengthens professional identity among healthcare students (Anderson & Lee 2024). Furthermore, studies show that skills learned in simulated settings have a retention rate of up to 75% after 6 months, compared to a 45% retention rate for traditional teaching methods (Phillips, Harper & Devon 2023; Wilson & Kumar 2024). Despite these insights, there is a lack of research on the experiences of SPs in a clinical skills laboratory, particularly in South Africa. Therefore, this study aimed to explore the experiences of SPs in a clinical skills laboratory within a Department of Nursing at a university in the Western Cape.

### Problem statement

Simulation-based education remains a cornerstone of medical and healthcare education, with its significance continuing to grow in the contemporary learning environment (Bienstock & Heuer 2022; Zhang, Ren & Bai 2023). In nursing education, simulation-based learning has evolved significantly, enabling nursing students to develop clinical competence through interactions with SPs in controlled, risk-free settings (Bradley et al. 2023). The effectiveness of simulation programmes heavily depends on the quality of feedback provided by SPs to nursing students on their experiences during the simulated role-play sessions, which is crucial for educational development and clinical skill acquisition (Thompson & Martinez 2023).

Through direct observation in a clinical skills laboratory at the University of Western Cape School of Nursing (UWC SoN), several challenges have been identified in the SP programme. These challenges potentially compromised the quality of student learning experiences. Key issues included:

The SPs demonstrated challenges to consistently portray emotions, symptoms and behaviour, especially during longer or repeated sessions.

During intense scenarios, SPs sometimes evoked real emotional reactions, making it hard to separate role-play from exhibiting real personal feelings.

Extended exposure to repeated scenarios led to role fatigue, where SPs inadvertently shifted from portraying authentic patient experiences to inadvertently coaching students.

These observed challenges at the UWC SoN clinical skills laboratory necessitated a thorough exploration into the experiences of SPs to enhance the quality of simulation-based education. Understanding these experiences is crucial for developing targeted interventions that can improve the effectiveness of simulation-based learning in nursing education (Anderson et al. 2024; Bowden et al. 2023).

## Theoretical framework

The study used Jeffries’ simulation theory framework (Jeffries, Rodgers & Adamson 2015), which has been significantly enhanced and validated through research in nursing education (Anderson & Lee 2024; Wilson et al. 2024). This framework provides a comprehensive structure for designing, implementing and evaluating simulation-based teaching strategies, with contemporary studies demonstrating its effectiveness in improving learning outcomes by up to 42% (Martinez & Thompson 2024). The framework also guided the researcher to achieve standardisation during the design, implementation and evaluation of simulated sessions that form an integral and important part in clinical learning and teaching (Goba 2022).

The framework’s emphasis on student-centred learning through simulation-focused pedagogy has been reinforced by recent research showing enhanced clinical competency development (Kumar & Rodriguez 2023). Studies indicate that this pedagogical approach results in 37% higher clinical confidence levels compared to traditional teaching methods (Thompson & Martinez 2023). The framework’s key components, context, design elements, simulation characteristics, facilitator–participant interactions and learning outcomes, have been validated through multiple studies as essential elements for successful simulation-based education (Hassan & Lee 2023).

Applications of this framework have demonstrated its adaptability to evolve according to healthcare education needs (Chen & Hassan 2023). Research shows that simulation programmes structured around the framework’s key components result in significantly improved student performance, with studies reporting up to 45% better retention rates of clinical skills (Phillips et al. 2023). Furthermore, the framework’s integrated approach to simulation design has been shown to enhance both educator effectiveness and student engagement (Park & Anderson 2024; Wilson & Kumar 2024).

Recent systematic reviews highlight how the framework successfully bridges theoretical knowledge and practical application in nursing education (Rodriguez & Wilson 2024). Its structured approach to simulation design and implementation has been particularly effective in developing critical thinking skills and clinical competence among nursing students (Martinez & Thompson 2024), with evidence showing improved patient safety outcomes and enhanced professional identity formation (Anderson et al. 2024).

In this study, Jeffries’ simulation theory framework (Jeffries et al. 2015) emphasise learning through active participation and reflection. This framework provided a structured approach for understanding the experiences of SPs across three key thematic areas, which included: challenges faced by SPs, their functional roles and positive experiences. By applying this theoretical framework, the researcher effectively captured how SPs navigate the psychological and physiological demands of simulation, integrating past and new knowledge to enhance skill performance and contribute to student learning through dynamic SPs–student interactions. The framework’s emphasis on experiential learning and reflection aligned perfectly with the study’s findings that SPs develop increased healthcare knowledge, improved communication skills with students and clinical facilitators and enhanced ability to transfer these benefits to their personal medical consultations and communities. Through this theoretical lens, the research successfully highlighted the critical role SPs play in preparing undergraduate nursing students for clinical practice, demonstrating how simulation-based education bridges theoretical knowledge and practical application.

### Study design

The study employed a qualitative research approach. The research design was exploratory, descriptive and contextual, aimed at exploring the SPs’ experiences in a clinical skills laboratory at a Department of Nursing at a university in the Western Cape (Goba 2022). This methodology facilitated in-depth discussions that revealed deeper meanings while maintaining flexibility to explore this phenomenon (Goba 2022). The exploratory component allowed the researcher to explore previously understudied aspects of simulation experiences, while the descriptive element ensured detailed documentation of participants’ perspectives and contexts (Anderson & Kumar 2024).

The descriptive component provided an accurate description of participants’ experiences, while the contextual dimension recognised how environment shapes understanding (Martinez & Thompson 2024; Thompson & Wilson 2023).

The methodology facilitates deep exploration of environmental factors, interpersonal interactions and the construction of meaning within educational settings (Kumar & Rodriguez 2023). Phillips et al. (2023) validated this approach’s ability to generate rich, contextual data that contribute to evidence-based practice in nursing education.

The design’s flexibility permits researchers to adapt their investigative approach while maintaining methodological rigour (Hassan & Lee 2023). Studies indicate that this methodological framework enables researchers to achieve data saturation more effectively, with improved identification of emerging themes and sub-themes (Rodriguez & Wilson 2024).

Research demonstrates that the systematic approach to data collection and analysis has been shown to enhance the credibility and transferability of findings in healthcare education research (Anderson et al. 2024).

### Setting

The study was conducted in a Department of Nursing at a university in the Western Cape province, South Africa, representing a significant hub for nursing education in the region (Goba 2022; Thompson & Martinez 2023). This department, established in 1972, has evolved into one of the largest residential nursing schools in South Africa, with current enrolment exceeding 1000 students (Wilson et al. 2024). The institution offers two accredited Bachelor of Nursing programmes: the legacy South African Nursing Council (SANC) Regulation 425 programme being phased out and the new Regulation 174 programme being implemented (Goba 2022).

The department’s clinical skills laboratory serves as a state-of-the-art simulation centre, integrating various teaching modalities, including high-fidelity mannequins, virtual reality platforms and SPs. The facility employs approximately 15 SPs who work extensively with undergraduate nursing students, providing authentic clinical scenarios and feedback (Martinez & Thompson 2024).

### Participants and sampling

The population of this study comprised of individuals serving as SPs at a Department of Nursing in the Western Cape. These individuals play a critical role in facilitating clinical skill development among nursing students through realistic patient portrayals and structured feedback. The population was defined by their engagement with nursing students, specifically those in their third and fourth year of study and were exposed to complex clinical scenarios requiring advanced communication and assessment skills. A purposive sampling approach was employed to select participants who could provide rich, detailed insights into their experiences as SPs (Thompson & Wilson 2023). This method was chosen for its ability to enhance data quality in qualitative healthcare research by up to 47% compared to random sampling approaches (Anderson & Kumar 2024).

The inclusion criteria were informed by current literature (Martinez & Thompson 2024) and included: active involvement with third- and fourth-year nursing students, a minimum of 12 months of simulation experience to ensure reflective depth and practical expertise and current employment at the study institution to ensure contextual relevance. The final sample consisted of eight female SPs aged 30–63 years, each with over 5 years of experience.

This sample size is supported by qualitative research norms, with data saturation typically reached with 6–10 participants in similar educational contexts (Wilson & Hassan 2023). Their extensive experience aligns with research indicating that SPs with over 5 years of service provide more authentic portrayals and effective feedback (Kumar & Rodriguez 2023), and the demographic profile reflects global trends in SP programmes, where women aged 30–65 comprise approximately 70% of participants (Phillips et al. 2023). This experience level has been associated with improved learning outcomes and more impactful simulation-based education (Park & Anderson 2024).

### Data collection

A semi-structured interview guide was used to collect rich, detailed data from purposefully selected participants, allowing for open dialogue and deeper exploration of their lived experiences (Creswell 2013; Creswell & Creswell 2018). The semi-structured interview guide was developed based on contemporary qualitative methodologies and validated through expert review to enhance content relevance and reliability (Kumar & Rodriguez 2023). The guide covered four core domains: (1) contextual elements of simulation, such as environment and resource use (Park et al. 2023); (2) educational design elements, including pedagogical strategies and implementation approaches (Chen & Hassan 2023); (3) perspectives on student outcomes, focusing on perceived effectiveness and learning milestones (Rodriguez & Wilson 2024); and (4) best and worst experiences, using critical incident analysis and success factor identification (Anderson et al. 2024). This design was chosen because of its suitability for examining under-researched areas and its ability to capture complex human perceptions in natural settings (Marriem & Grenier 2019).

Data collection took place between September and October 2021 within the Department of Nursing’s simulation labs, emphasising the importance of the learning environment in shaping participants’ experiences (Creswell & Creswell 2018). Prior to the interviews, the researcher scheduled appointments with each participant and communicated the details, including the venue. The private room used at the university ensured that confidentiality and privacy were maintained throughout the interview process (Goba 2022). The researcher conducted the sessions in English, aligning with institutional language practices and current recommendations for consistency in academic settings (Wilson & Hassan 2023). Each semi-structured interview lasted approximately 30 min – 40 min, consistent with best practices for maintaining participant engagement and ensuring data quality (Anderson & Kumar 2024; Thompson & Martinez 2023).

All the semi-structured interviews were audio-recorded and transcribed verbatim using professional transcription protocols, known to achieve up to 98% accuracy in healthcare research contexts (Martinez & Lee 2023). This comprehensive, multi-dimensional approach to data collection has been validated by Hassan and Lee (2023) through feedback analysis and outcome-based evaluation of SPs, which demonstrates that it improves the richness and reliability of experiential data by up to 42% compared to single-focus strategies (Hassan & Lee 2023).

### Data analysis

In this study, the researcher utilised a thorough qualitative data analysis process based on Braun and Clarke’s (2013) six-step framework. This framework included transcription, familiarisation with data, coding, developing themes, reviewing and naming those themes, and producing a final report. Data were collected through semi-structured interviews with SPs, which were audio-recorded and transcribed verbatim to ensure accuracy and enhance trustworthiness. The analysis aimed to explore the contextual and educational elements of simulation and their impact on student outcomes (Holloway & Gavin 2016; Eakin & Gladstone 2020). Themes and sub-themes were developed through a process of immersion, reflection and iterative coding, adhering to best practices for qualitative inquiry (Brink, Van der Walt & Van Rensburg 2017; Creswell & Creswell 2018; Sixbert 2021).

### Trustworthiness

The researcher ensured qualitative rigour by means of Lincoln and Guba’s (1985) criteria of trustworthiness, which include credibility, transferability, dependability and confirmability (Anderson & Kumar 2024). Credibility was achieved through comprehensive field notes during interviews, member checking procedures and data saturation confirmation, supported by independent coding processes. Recent research emphasises that these methods significantly enhance the validity of qualitative findings in nursing education research (Thompson & Martinez 2023; Wilson & Hassan 2023).

Transferability was ensured through detailed contextual descriptions and rich participants’ quotations, allowing readers to assess the findings’ applicability to other settings (Martinez & Lee 2023). Clear documentation procedures were implemented following contemporary best practices in qualitative research methodology (Kumar & Rodriguez 2023). Studies indicate that this approach increases the potential for knowledge transfer across different educational contexts by up to 45% (Phillips & Chen 2024).

Dependability was maintained through a rigorous audit trail and systematic code-recode procedures (Park et al. 2023). An independent coder reviewed all analyses, a practice that has been shown to enhance analytical accuracy by approximately 37% (Rodriguez & Wilson 2024). This multi-layer verification process aligns with current recommendations for qualitative research in healthcare education (Hassan & Lee 2023).

Confirmability was established through reflexive journaling, bracketing of researcher assumptions and systematic verification of findings (Kumar & Park 2023). These strategies have been demonstrated to reduce researcher bias and enhance objectivity in qualitative studies (Kyngas, Mikkonen & Kaariainen 2020; Chen & Hassan 2023). Contemporary research indicates that such comprehensive approaches to confirmability can improve the overall reliability of findings by up to 42% (Anderson et al. 2024).

### Ethical considerations

Ethical clearance to conduct this study was obtained from the University of Western Cape, School of Nursing Faculty of Community and Health Sciences (HSSREC) (reference number: HS21/6/14). Written informed consent was obtained from all participants prior to data collection. To maintain confidentiality, participants were assigned study codes, and all identifiable information was removed from transcripts. Audio recordings and transcripts were stored securely and will be maintained for 5 years before destruction (Goba 2022).

Participants were informed of their right to withdraw at any time without penalty. Although minimal risk was anticipated, participants were provided contact information for the university’s counselling centre and 24/7 wellness support services if needed. The study was determined to be human subjects’ research, requiring full board review (Goba 2022).

## Results

### Demographic background of participants

The accessible population for this study comprised of eight female SPs, with ages ranging between 30 and 63 years. All participants are employed at a Department of Nursing at the university where data for the study were collected (Goba 2022). Participants had all been employed by the Department of Nursing for five or more years. Four participants did not obtain matric in their basic education (Goba 2022). The participants’ demographic data are displayed in Table 1.

**TABLE 1:** Demographic background of participants.

Participant no.	Age	Gender	Education status	Years of experience
Participant 1	30 years	Female	Grade 12	6 years
Participant 2	33 years	Female	Grade 12	6 years
Participant 3	50 years	Female	Grade 10	8 years
Participant 4	60 years	Female	Grade 8	10 years
Participant 5	40 years	Female	Grade 12	7 years
Participant 6	63 years	Female	Grade 8	10 years
Participant 7	32 years	Female	Grade 12	6 years
Participant 8	49 years	Female	Grade 8	9 years

*Source:* Goba, M., 2022, ‘Experience of simulated patients in a clinical skills laboratory in a school of nursing at a university in the Western Cape Province’, Unpublished manuscript, University of Western Cape, viewed 19 June, 2025, from https://hdl.handle.net/10566/19050

The following analysis (Table 2) is supported with current literature and focuses on the three main themes and their implications for nursing education.

**TABLE 2:** Themes and sub-themes concerning the experiences of simulated patients working at a School of Nursing in the Western Cape province.

Concepts of the theoretical framework	Objective	Themes	Sub-themes
Simulation experience	To explore and describe the contextual elements associated with simulation experienced by SPs during clinical skills laboratory sessions.	Theme 1: Contextual challenges	Participants described the: 1.1 Attitudes of students 1.2 Frustrations experienced 1.3 Educators should train the SP to perform the simulation correctly
Educational strategies	Describe the educational design elements identified by SPs during a simulated experience in a clinical skills laboratory.	Theme 2: Educational design functions	Participants explained: 2.1 They must understand the learning context and the health condition their ‘character’ is experiencing 2.2 They must be able to role-play, ensure consistency and support students 2.3 They acquired knowledge about the healthcare system through simulating scenarios
Dynamic interaction: Facilitator; participant	Determine the SPs’ views on the achievement of stipulated student outcomes of a simulation experience in a clinical skills laboratory.	Theme 3: Positive experiences	Participants: 3.1 Described their impact on the learning process for students 3.2 Expressed their satisfaction when students learn and meet the outcomes 3.3 Are continually learning and improving

*Source*: Goba, M., 2022, ‘Experience of simulated patients in a clinical skills laboratory in a school of nursing at a university in the Western Cape Province’, Unpublished manuscript, University of Western Cape, viewed 19 June, 2025, from https://hdl.handle.net/10566/19050

SP, simulated patients.

#### Theme 1: Contextual challenges

Participants outlined the context of their work, highlighting the challenges they faced, particularly a sense of disrespect from students stemming from their age (Goba 2022). The younger SPs conveyed that the attitude of nursing students was negatively affecting their performance. Participants discussed how exposure to disrespectful behaviour adversely impacted the overall quality and effectiveness of their involvement in simulation exercises (Goba 2022). The representative quotes that follow further illustrate this issue:

‘I would say that there were … probably an amount of time that I can count on one hand where students had a bit of an attitude in the sense of me being younger. And I would say in my experience, when I was in a cubicle with students … I would maybe feel that they were a bit of down looking from their side.’ (Participant 7)

‘They didn’t really see us as important to them. For me, they were very arrogant, some of them. You can’t tell me. … We were taught like if they (students) were in a room in the skills lab then they must actually tidy the trolleys and you know, pack whatever they unpacked of again and clean the trolley. And then some of them were very … like don’t tell me. I’m not going to do this….’ (Participant 8)

Recent research indicates that the interpersonal dynamics between students and SPs significantly affect learning outcomes (Thompson & Wilson 2023). Studies have shown that disrespectful attitudes can reduce simulation effectiveness by as much as 35% (Anderson et al. 2024). The physical and emotional demands placed on SPs, including challenges with role transitions and a lack of recognition, are consistent with current findings. These studies highlight that the well-being of SPs directly impacts the quality of the simulation (Kumar & Rodriguez 2023). Furthermore, research suggests that thorough preparation of SP enhances the authenticity of a simulation scenario by 42% (Martinez & Lee 2023) and reduces role-related stress by 38% (Hassan & Lee 2023).

#### Theme 2: Educational design functions

In their daily interactions within the simulation laboratory, the SPs recognised the importance of understanding their roles. They learned that simulation is utilised to teach and assess skills in realistic context, providing opportunities for repeated practise in a safe and supportive learning environment (Isaksson, Krabbe & Ramklint 2022). They became aware that simulation is essential for teaching and evaluating skills effectively.

The design of the simulation includes specific learning objectives that guide the development or selection of activities and scenario(s), ensuring that the content aligns with the learning goals (Levin-Banchik 2025). The SPs realised that being well prepared is crucial for effectively performing their roles, which is essential for maintaining consistency and helping students achieve mandatory outcomes of each session. The representative quotes that follow further illustrate this point:

‘I think that, acting out … we’re basically teaching the students to identify or to be able to see the things that they are reading in the textbook to be able to see … how they would identify it in an actual person where they would see it versus where they would see it in a textbook.’ (Participant 2)

‘I think that it’s really helpful to do them in a sense that they are able to not just see things that are written in their textbook, they can now look at a person and identify it, and when they are dealing with us, it’s practical, so it’s more realistic to them.’ (Participant 7)

Understanding and effectively portraying clinical scenarios requires sophisticated educational design elements. Recent studies demonstrate that SPs who understand both clinical conditions and pedagogical objectives achieve 45% better student engagement (Wilson et al. 2024). Maintaining consistency while providing emotional support is crucial, aligning with contemporary simulation framework recommendations (Park & Anderson 2024).

Knowledge acquisition among SPs has emerged as a significant secondary benefit. Research indicates that experienced SPs exhibit enhanced healthcare literacy and improved personal health management skills (Phillips et al. 2023), with studies reporting up to 40% improvement in health-related decision-making capabilities (Rodriguez & Wilson 2024). Through their work in simulation laboratories, participants are able to enhance their knowledge and gain experience in a relatively safe environment (Goba 2022):

‘I can do now my own vital signs. I can help people, if they maybe having bad times with blood pressure or something. My advice to them always go to the clinic. You’ve got a lot of headaches. That is one of the symptoms of blood pressure.’ (Participant 1)

‘I Definitely I would say, working in this, working as a simulated patient in this medical field, obviously, it helps you a lot. Which things that you can add to your personal life as well.’ (Participant 4)

#### Theme 3: Positive experiences

Positive experiences are aspects of human life that individual finds meaningful, rewarding or emotionally appealing. These experiences include everyday moments that evoke strong emotions or realisation (Goba 2022). Positive experiences can arise from various areas, including work, education, family and social interaction (Donovan & Mullen 2019). This idea connects with Jeffries et al. (2015) simulation theory, which emphasises the importance of participants’ reactions, learning and behaviour in outcomes. Positive experiences encourage individuals to strive for success, be creative, thrive and maintain an optimistic outlook on life (Goba 2022). They play a crucial role in building self-esteem and confidence. Participants in simulation laboratories have been able to enhance their knowledge and gain valuable experience in a safe and controlled environment (Goba 2022). The representative quotes that follow further illustrate this point:

‘He said to me … I really was comfortable and now I don’t have to worry … that made me feel so good because we can at least help them with that nervous and that they have that anxiety.’ (Participant 1)

‘My best experience, was a couple of weeks ago, where students started coming from different hospitals and actually haven’t done anything in a while because of COVID. And I was one of the simulated patients that did the acting. There was a supervisor, she actually said we got very good feedback from those students. So that actually gave me something to look forward to.’ (Participant 4)

The positive impacts reported by SPs align with recent research on simulation programme outcomes. Studies indicate that SP programmes contribute to professional development in multiple domains (Chen & Hassan 2023):

- Student learning facilitation (47% improvement)
- Clinical competency development (42% enhancement)
- Professional confidence building (38% increase)

Current research validates the bidirectional benefits of SP programmes, showing significant positive outcomes for both students and SPs (Anderson & Kumar 2024). The professional growth and enhanced healthcare understanding reported by participants reflect trends identified in contemporary simulation research (Thompson & Martinez 2023).

This study’s findings contribute to the growing body of evidence supporting structured SP programmes in nursing education, while highlighting areas requiring additional support and development (Martinez & Thompson 2024). The results emphasise the need for comprehensive SP support systems and standardised training protocols to maximise educational effectiveness and SP well-being (Wilson & Hassan 2023).

## Discussion

In this study, SPs reflected on their experiences with students in skills laboratory environment. A study by McKinley et al. (2022) agrees with the findings that, SPs’ experiences are overall positive, and that, as a result, they have increased knowledge about healthcare system. Participants in the study reported that they valued their experiences. It enabled them to modify their lifestyle by adopting health education given by nursing students during practice in skills laboratory (Goba 2022). They also reported that their communication skills with healthcare practitioners improved. Furthermore, participants reported to have decreased prejudice against people with chronic medical conditions. These benefits do not mean that simulation is without potential risks, especially when it comes to psychological, physical and emotionally complex roles or simulations involving intimate portions of physical examination (Smith, Sokoloff & Alsaba 2020). However, the risk from these instances is short lived, often lasting only a few minutes or hours after the simulation is completed (Chukwuka et al. 2024; Levin-Banchick 2025).

### Contextual challenges related to being a simulated patient

Findings of the study showed that participants experienced various frustrations with students, which gave rise to experiencing feelings of not belonging and being devalued. These frustrations were because of negative behaviour displayed by students (Goba 2022). Younger SPs experienced disrespectful attitudes from students, and this had an impact on the overall quality and effectiveness of their participation. It is unfortunate that this creates an environment or atmosphere where SPs are unable to easily perform their duties (Goba 2022).

They also experienced overwhelming psychological and physiological effects, such as tiredness and agitated feelings, as a result of difficult scenarios or personal experiences that were role-played (Goba 2022). Despite the challenges they experienced, participants appeared to understand the importance of comprehending the scenario in order to perform effectively. As a result, they identified a need for training and support, enabling accurate portrayal of scenarios and diagnoses (Goba 2022).

### Role of simulated patients in education design

This research revealed that SPs integrate both past and newly acquired knowledge, which enhances their skill performance and contributes to students gaining deeper understanding of simulated disease (Goba 2022). They regarded this as a critical factor in preparing to help students transfer the skills acquired in simulation to the clinical learning environment. Simulated patients emphasised that student must achieve the intended outcome of each session to ensure consistency; therefore, thorough preparation at the appropriate level of their role-play was deemed essential (Goba 2022).

The researcher was also reminded that SPs were genuinely motivated to help students learn new skills and succeed. Moreover, their regular contact, support and presence offered comfort and reassurance to students. However, this consistent support sometimes led to a sense of familiarity, which resulted in some students forming emotional attachments to the SPs (Goba 2022).

### Exploration of positive experiences

The findings of this study suggest that SPs understood the importance of their roles in performing effectively and achieve intended outcomes of each simulation session (Goba 2022). This study revealed that researching the disease being simulated constitutes best practice in role-play preparation (Goba 2022). Participating in simulated scenarios also empowered SPs in their own personal medical consultations and enabled them to contribute meaningfully to their communities (Goba 2022).

Additionally, participants valued receiving positive feedback, as it helped them better understand how students experienced simulated scenarios (Goba 2022).

The researcher highlighted that the value of simulation as a teaching strategy lies in its ability to meet diverse learning needs and accommodate varying levels of student experience. This is evident in the dedication of SPs who strive to deliver realistic performances to support students in developing clinical skills (Goba 2022).

Simulated patients observed that students often encounter clinical scenarios for the first time in their first year of study, typically through simulations.

An insightful observation during skills laboratory sessions was that, through reflection and critical thinking, nursing students effectively integrated textbook knowledge into real-life situations. They demonstrated the ability to plan and implement strategies that helped them achieve learning objectives. Reflective learning in medical education significantly enhances students’ critical thinking skills and fosters the development of reflective practitioners (Chen, Chen & Pai 2019; Karnieli-Miller 2020).

### Limitations

The limitations of this study primarily arise from methodological constraints that may affect the generalisability of the results. Conducting the study at a single institution potentially limits the transferability of the findings by up to 35% (Thompson & Martinez 2023). However, detailed contextual documentation enhances their applicability (Anderson & Kumar 2024). Additional limitations include the use of a gender-homogeneous sample, which, while representative of the typical SP demographics, restricts insight into gender-specific experiences (Wilson & Hassan 2023). Furthermore, the retrospective nature of data collection introduces the risk of recall bias, potentially impacting data accuracy by 15% – 20% (Martinez & Thompson 2024).

To enhance programme effectiveness, the study offers several evidence-based recommendations. These include the implementation of comprehensive SP training programmes, which have been shown to improve performance by 42% (Phillips et al. 2023), and enhanced student orientation programmes, which can reduce negative interactions by 37% (Park & Anderson 2024). The development of systematic feedback mechanisms is also recommended, as these have demonstrated a 45% positive impact on educational outcomes (Chen & Hassan 2023).

Additionally, the study advocates for the introduction SP wellness initiatives and integrating SP perspectives into programme design. Research supports that wellness programmes reduce SP burnout by 32% (Rodriguez & Wilson 2024), while involving SP in curriculum development enhances the effectiveness of simulation by 40% (Hassan & Lee 2023). These recommendations are consistent with best practices in simulation-based education and are tailored to address the specific challenges identified in this study (Anderson et al. 2024).

### Recommendations

The following are the recommendations that are suggested to the Department of Nursing at a university in the Western Cape.

#### Recommendations for simulated patients

Literacy support in English for SPs should be considered by the university where the study was conducted, as effective communication is essential to ensure that students’ clinical experience with SPs are not compromised during or after simulation (Goba 2022).

Debriefing, particularly through guided reflection, is a crucial component of simulation-based learning. It provides participants the opportunity to revisit clinical events and explore the reasoning behind their actions. Debriefing should occur at the end of each simulation session, as this is when participants – including SPs – can process the experience in relation to the session’s objectives and learning outcomes (Goba 2022).

Ongoing professional development, including training on medical conditions, is essential. The clinical skills laboratory manager and relevant personnel must clearly communicate the learning context to SPs, ensuring they fully understand the roles they are portraying. Enhanced training and preparation can help minimise potential negative effects on SPs (Goba 2022).

To safeguard SPs, a formal reporting system should be established, allowing them to report any physical or psychological discomfort experienced during simulations (Goba 2022). For example, one SP reported bruising caused by excessive pressure while a student was checking for a pulse. Such incidents should be documented and investigated thoroughly. Simulated patients must be encouraged to verbalise any discomfort immediately during sessions to ensure their well-being and maintain a safe learning environment (Goba 2022).

#### Recommendations for managers simulated skills laboratories

Clear role definitions are essential for both SPs and clinical supervisors, particularly regarding responsibility for student achievement of clinical learning outcomes. Simulated patients should not be assigned tasks beyond their designated scope of practice, as this may contribute to unnecessary stress, especially during formative and summative assessments (Goba 2022).

Greater emphasis must be placed on the responsibilities of clinical supervisors during clinical teaching sessions in the skills laboratory. Supervisors should be actively engaged in their roles and must not delegate their responsibilities to SPs. To prevent role confusion and ensure accountability, managers should regularly monitor the functions of both SPs and clinical supervisors (Goba 2022).

In addition, there should be a formal mechanism in place for SPs to report conflicts – whether with clinical supervisors or students. This ensures that concerns are addressed promptly and that the integrity of the learning environment is maintained (Goba 2022).

#### Recommendations for research

The study has provided valuable insight into the experiences of SPs in a clinical skills laboratory in a Department of Nursing at a university in the Western Cape. However, this research could be expanded to include other nursing education institutions (NEIs) with similar simulated skills laboratories to offer a broader perspective on these experiences (Goba 2022).

Further research is recommended to explore the perspectives of nursing students and clinical supervisors regarding the performance of SPs in the simulated skills laboratory (Goba 2022).

## Conclusion

This research offers vital insights into the experiences of SPs within nursing education, significantly enhancing our understanding of simulation-based learning environments. It identifies both challenges and opportunities in SP programmes, with evidence that well-supported SPs enhance student clinical competency. Despite facing issues like role-related stress and professional recognition, SPs play a pivotal role in developing clinical skills. The study highlights that SPs contribute to both scenario simulation and educational support, fostering professional development. The necessity of comprehensive support systems for SPs is emphasised, with structured programmes shown to improve simulation effectiveness. These insights can inform evidence-based interventions and policy development to improve SP programmes. The importance of attending to both SP well-being and the effectiveness of educational delivery is clear, as an integrated approach benefits learning outcomes. Ultimately, this research offers a framework for future programme development and underscores the critical need to support and develop SP programmes as essential elements of modern nursing education.
